# Effects of Extrinsic and Intrinsic Motivation on Selective Activation of Shoulder Girdle Muscles During the Barbell Bench Press Exercise

**DOI:** 10.3390/jfmk9040218

**Published:** 2024-11-02

**Authors:** Katarzyna Strońska-Garbień, Artur Terbalyan, Mariola Gepfert, Robert Roczniok, Miłosz Drozd, Artur Gołaś, Adam Zając

**Affiliations:** Department of Sports Training, Institute of Sport Sciences, The Jerzy Kukuczka Academy of Physical Education, 40-065 Katowice, Poland; k.stronska@awf.katowice.pl (K.S.-G.); a.terbalyan@awf.katowice.pl (A.T.); m.gepfert@awf.katowice.pl (M.G.); r.roczniok@awf.katowice.pl (R.R.); m.drozd@awf.katowice.pl (M.D.); a.zajac@awf.katowice.pl (A.Z.)

**Keywords:** electromyography, resistance training, selective recruitment

## Abstract

**Background/Objectives:** This study aimed to investigate the effects of intrinsic and extrinsic motivation on selective muscle activation of the shoulder girdle during the barbell bench press. Specifically, this research focused on how attentional focus on individual muscles, such as the anterior deltoid (AD), pectoralis major (PM), and triceps brachii long (TBL), could influence their electromyographic (EMG) activity during the exercise. **Methods**: Twelve male participants, with at least five years of strength training experience, performed bench press exercises under two conditions: with extrinsic motivation (no specific focus on muscle activity) and with intrinsic motivation (internal focus on specific muscles). Surface electromyography (sEMG) was used to measure muscle activity during three sets of bench presses at 60% of one repetition maximum (1RM). Participants were instructed to focus on the activation of specific muscles in a randomized sequence. **Results**: The intrinsic motivation condition significantly increased muscle activation compared to extrinsic motivation. Electromyographic activity of the AD, PM, and TBL muscles was notably higher when participants focused their attention on these muscles. AD activation increased from 71.78 ± 11.13%MVC (extrinsic) to 88.03 ± 8.84%MVC (intrinsic) (*p* = 0.0019), while PM and TBL activation also demonstrated significant increases under intrinsic focus. **Conclusions**: The study concludes that intrinsic motivation, or an internal focus on specific muscle activation, can significantly enhance EMG activity in target muscles during the bench press exercise. This finding has important implications for resistance training and rehabilitation, where focused muscle activation can be utilized to improve training outcomes and muscle engagement.

## 1. Introduction

The bench press is one of the most commonly employed resistance exercises for upper body development. Due to its effectiveness in enhancing the strength and power of the shoulders and chest, it is widely utilized by athletes across various sports disciplines [1]. Successful execution of the bench press involves lowering the barbell to the chest, followed by extending it to a fully locked-out position. The movement can be divided into two distinct phases: the eccentric (lowering) and concentric (ascending) phases. Performance in the bench press is typically assessed by the maximum load that can be lowered to the chest and then pressed to full elbow extension.

In exercise biomechanics, the phenomenon of muscle activation is a key indicator of resistance training efficacy. Recent research has indicated that with appropriate guidance, individuals can voluntarily influence the activation of specific muscles during training [2]. This can be achieved by relaxing or activating specific muscles, or by selectively inhibiting or activating individual motor units through conscious motor control. It has been demonstrated that intense resistance exercise results in relatively high levels of muscle activation [3,4]. Over an extended training period, this leads to an increase in muscular strength [4] and can also improve athletic performance, musculoskeletal fitness, and body shape [5]. Multi-joint exercises represent the primary means of developing muscular strength. Many compound resistance exercises engage multiple muscle groups and are susceptible to alterations in muscle control. Extensive literature indicates that it is feasible to perform submaximal actions by selectively activating specific muscles while reducing the contribution of other muscles engaged in the same task. This voluntary control of muscle activation patterns offers a range of potential benefits for exercise and therapeutic application [6].

The properly executed bench press is characterized by a highly reproducible movement pattern, customized to align with an individual’s anthropometric features. Various methodological adjustments, such as grip width, bench angle, and variations in incline, are employed to optimize technique. The inclusion of dumbbells, unstable surfaces, and different bar types serves to enhance both balance and coordination. A number of studies investigating muscle strength topography highlight the contribution of specific muscle groups to overall strength output. Neuromuscular recruitment has predominantly been evaluated through EMG amplitude to measure the impact of exercise load as an external stimulus for muscular hypertrophy. EMG analysis provides five fundamental categories of data: muscle activity, the magnitude of muscle activation, the temporal pattern of muscle activation and deactivation, the extent of muscle engagement, and the level of muscle fatigue. The tonic neurophysiological behavior of motor units during contraction, particularly in relation to activation intensity, must be carefully considered in initial assessments.

The barbell bench press exercise engages three primary muscle groups: the pectoralis major (PM), the anterior deltoid (AD), and the triceps brachii long (TBL) [7,8]. The level of electromyographic activity exhibited by these muscles during the bench press is dependent on several factors. Lehman et al. demonstrated that narrowing the grip width resulted in increased activation of the triceps brachii muscle [9]. In contrast, Rebecca and Cook [2] found no significant differences in muscle activity when different hand positions on the barbell were considered. Additional factors that affect muscle activity during the bench press exercise include the angle of the bench [10] and resistance training experience [11]. Moreover, the bench press is a key exercise in both competitive sports and general fitness, making it a prime candidate for investigating the neuromuscular adaptations associated with different motivational strategies [10]. Athletes, strength coaches, and rehabilitation specialists can benefit from this knowledge by implementing specific motivational techniques to optimize training efficiency, muscle engagement, and long-term development.

Focus of attention is considered an integral strategy in the field of motor learning. Focus of attention can be defined as what the subject thinks about while performing a movement or activity [12,13]. There are two basic strategies for focusing attention while performing a task: internal and external. Internal focus involves thinking about a particular body movement while performing, while external focus involves shifting performance-oriented focus to the environment.

The bench press exercise is also influenced by both intrinsic motivation, which is focused on the movement itself, and extrinsic motivation, which is focused on the outcome [14,15,16]. Concentration represents a crucial aspect of the training process in sports. It constitutes a significant factor in the development of speed in a variety of sporting activities and is also of importance during resistance training. Focused attention on the performance of motor skills has an impact on performance and the acquisition of motor skills [17].

Range of motion (ROM) is a significant factor influencing strength and hypertrophic gains during resistance exercise [18]. Studies have demonstrated that exercises with full ROM result in higher muscle activation, sEMG, and isometric fatigue compared to partial ROM [19]. Tsoukos et al. compared strength and power combined with sEMG data in the bench press between three different ROMs (full, top, bottom) for the PM, AD, and TB muscles. The sEMG variables (RMS, iEMG) increased at the end of each set in all ROMs for all muscles tested. However, the sEMG variables of the TB muscle showed different responses between ROMs, with RMS and iEMG being higher in FULL and TOP and lower in BOTTOM (FULL > TOP > BOTTOM). Also, the median frequency in TB and AD muscles was higher in TOP and FULL ROM compared to BOTTOM at first repetitions [20].

The internal structure of the barbell bench press exercise has been studied extensively [7,8,10,11,21,22]. Nevertheless, there is still a paucity of information pertaining to the electromyographic analysis of the shoulder girdle muscles in the context of an additional attentional focus on individual muscle groups. In recent years, there has been a growing interest in the mechanisms of selective muscle activation during resistance exercises such as bench press [12,14,23,24]. This has led to a deeper exploration of how different types of motivation—intrinsic, where the focus is on the movement and muscle engagement, and extrinsic, where the focus is on the overall outcome—affect muscle activation patterns. Understanding how athletes can consciously control muscle activation has significant implications for improving both performance and rehabilitation outcomes [3]. Studies have shown that attentional focus can enhance muscle recruitment, which is essential for achieving specific training goals such as hypertrophy, strength gain, or injury prevention [2,23]. By examining the effects of intrinsic versus extrinsic motivation, this study seeks to determine how these psychological factors can modulate EMG activity during the bench press exercise, particularly in the shoulder girdle muscles, which play a critical role in upper body performance. The objective of this study was to analyze the electromyographic activity of the shoulder girdle muscles in response to intrinsic motivation, defined as the internal focus of attention on the movement of specific muscles. We hypothesized that an intrinsic focus of attention on specific shoulder girdle muscles during the barbell bench press would significantly increase their EMG activity compared to an extrinsic focus.

## 2. Materials and Methods

### 2.1. Experimental Design

The measurements were performed in the Laboratory of Muscular Strength and Power at the Academy of Physical Education in Katowice. Four weeks before the experiment, the participants were familiarized with the study design in order to improve their flat bench press technique.

### 2.2. Participants

A total of 12 male participants were included in the study. The G*power (v3.1.9.6, Kiel University, Kiel, Germany) software was used to determine a priori sample size. To achieve an effect size level of 0.6 (α = 0.05; power = 0.96), it was necessary to recruit 12 participants. The participants were selected purposely and were required to have a minimum of five years of experience in strength training. Each subject demonstrated a proper barbell bench press technique, with a BP 1 repetition maximum (1RM) of 95 ± 30 kg. The mean age, weight, and height of the participants were 28 ± 7.6 years, 88.1 ± 10.2 kg, and 181.7 ± 11.3 cm, respectively. The participants were asked to abstain from any form of resistance exercise for a minimum of 72 h prior to the experiment to prevent any fatigue-related effects on their performance. They were also informed about the experimental procedures, the study’s potential risks and benefits, and the option to withdraw from the experiment at any stage. Each participant provided written consent for their involvement in the study. The protocol was approved by the Bioethics Committee for Scientific Research at the Academy of Physical Education in Katowice.

### 2.3. The Determination of 1RM (One-Repetition Maximum)

The determination of 1RM was performed according to the protocol by Tillaar and Saeterbakken [25]. The 1RM load was determined based on the self-reported values by the athletes. The reported 1RM data on maximal lifts was acquired from the previous three months. The rest intervals between sets equaled 5 min to secure full recovery and avoid potential effects of fatigue. When the self-reported 1RM was successful, a trial with an additional load of 2.5–5 kg was performed. When the initial trial was unsuccessful, the weight was decreased by 2.5–5 kg. A total of two to three trials were performed per athlete. The tempo of movement of all bench press exercises was controlled by a metronome (Korg MA-30, Korg, Melville, NY, USA).

### 2.4. Procedures

On the day of the experiment, the subjects performed the bench press protocol and 3 s maximum isometric MVC contractions in order to normalize the results. The warm-up consisted of two distinct phases: a general and a specific part. In the initial phase, the participants engaged in a 5 min continuous exercise on a hand-held bicycle ergometer, maintaining a heart rate of approximately 130 beats per minute. This was followed by a set of strength exercises for the upper body, performed without external load. The specific part of the warm-up consisted of three bench press sets with the load adjusted accordingly to perform 15, 10 and 5 repetitions.

#### 2.4.1. Experimental Protocol

This study was conducted in two distinct sequential stages. In the first stage of the study, the subjects were asked to perform one 1 set of 3 repetitions of the bench press with a load of 60% of 1RM. The choice of 60% 1RM was selected because it represents a submaximal load, which allows for investigating the effects of muscle activation without reaching the fatigue levels associated with higher intensities. This load is sufficient to elicit significant muscle activation, as observed through EMG while allowing for controlled, repeatable measurements [16,26]. In stage two, the subjects performed 3 sets of 3 repetitions of the bench press with a load of 60% 1RM and internal attention, successively focusing on the pectoral, shoulder, or triceps muscles in each set. Prior to and during each set of the bench press in stage 2, the subject was verbally instructed to ensure that their attention was focused on the movement of a specific muscle (internal focus). In the initial set of the test, the subject’s attention was directed towards the pectoral muscle. In the subsequent set, the focus shifted to the shoulder muscle, and in the last set, the subject concentrated on activating the triceps brachii muscle. The rest intervals between the sets were 3 min, and those between particular stages equaled 5 min.

The dominant upper limb was subjected to examination. For all participants, this was the right limb. To ensure consistency and comparability across participants, each individual was instructed to perform two 3 s maximum isometric MVC tests for each muscle (PM, AD, TBL) and for all muscles collectively, with a load that did not allow the barbell to move (200% 1RM). The angle between the arm and forearm was 90° [27]. The mean peak value (PEAK) for each of the muscles was used as the basis for the statistical analysis of the %MVC achieved during the EMG assessment.

#### 2.4.2. Electromyography

An eight-channel Noraxon TeleMyo 2400 system (Noraxon USA Inc., Scottsdale, AZ, USA, 1500 Hz) was used for recording and analysis of biopotentials from the muscles. The activity was recorded for three muscles: PM, AD and TBlong. Before placing the gel-coated self-adhesive electrodes (Dri-Stick Silver circular sEMG Electrodes AE-131, NeuroDyne Medical, Newton, MA, USA), the skin was shaved, abraded and washed with alcohol. The electrodes (11 mm contact diameter and a 2 cm center-to-center distance) were placed along the presumed direction of the underlying muscle fiber according to the recommendations by SENIAM [28]. The EMG signals were sampled at a rate of 1000 Hz. Signals were bandpass filtered with a cut-off frequency of 8 Hz and 450 Hz, after which the root-mean-square (RMS) was calculated. All the electrodes were positioned on the dominant side of the body. The grounding electrode was placed on the connection with the triceps brachii muscle. Video recording was used for identification of the beginning and completion of the movement. After completion of all the tests in a single day, 2–3 s evaluations of Maximal Voluntary Contraction (MVC) of the prime movers in the bench press movement (AD, PM and TBlong) were performed according to the SENIAM procedure. These evaluations were performed in order to normalize electromyographic records. The analysis was based on peak activity during the bench press exercise from both the eccentric and concentric phases of the movement.

### 2.5. Statistical Analysis

All analyses were conducted using the Statistica 13.1 (StatSoft, Palo Alto, CA, USA) and PRISM 9.4.1 (GraphPad, Boston, MA, USA) software packages. The normality of the distributions was verified using the Shapiro–Wilk test, the Levene test was employed to verify the homogeneity of variances, and the Mauchly test was used to verify sphericity. The results were presented as means with standard deviations, standard errors, and 95% confidence intervals. A multi-criteria repeated-measures ANOVA was employed to ascertain the discrepancies between the variables under consideration. The effect sizes for the main effects and interactions were determined by partial eta squared (η^2^). The ES was classified as small (0.01 to 0.059), moderate (0.06 to 0.137), and large (>0.137). In the event of significant differences in the main effect or interaction, post hoc comparisons were conducted using Bonferroni’s post hoc test. The level of statistical significance for the differences between the types of loads and muscle sides was set at *p* < 0.05. Effect sizes (Cohen’s d) were also calculated. The ES was interpreted as large for d > 0.8, moderate for d between 0.8 and 0.5, and small for d < 0.5.

## 3. Results

**Figure 1:**
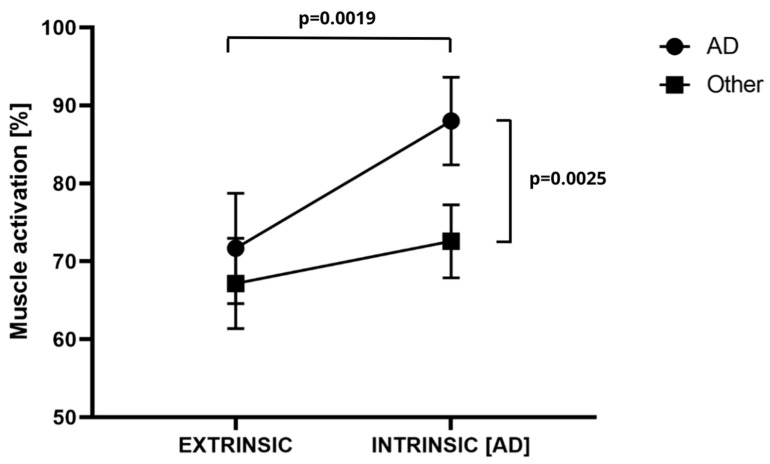
Muscle × extrinsic–intrinsic interaction AD (anterior deltoid) activity. *p*-test probability. Other—reference point in the form of the mean of the rest of the muscles outside of the intrinsic focus.

The results of the analysis of variance for AD revealed significant differences for the main effects of muscle (F = 9.11; *p* = 0.0048; η^2^ = 0.21), extrinsic–intrinsic (F = 19.02; *p* = 0.0001; η^2^ = 0.36), and the muscle × extrinsic–intrinsic interaction (F = 4.37; *p* = 0.037; η^2^ = 0.12). The activation of the AD muscle subsequent to motivation was found to be significantly higher than that observed at extrinsic (AD motivation 88.03 ± 8.84%MVC vs. extrinsic 71.78 ± 11.13%MVC; *p* = 0.0019; d = 1.56). No significant differences were observed between the extrinsic (67.18 ± 13.67 %MVC) and intrinsic (72.62 ± 11.08%MVC) measurements for the other muscles (*p* = 0.25). Activation following motivation for the AD muscle (88.03 ± 8.84%MVC) was significantly higher than for the other muscles (72.62 ± 11.08%MVC; *p* = 0.0025; d = 1.48). These results are also supported by the data illustrated in Figure 1. Basic descriptive statistics for dependent variables are presented in Table 1 below.

**Figure 2:**
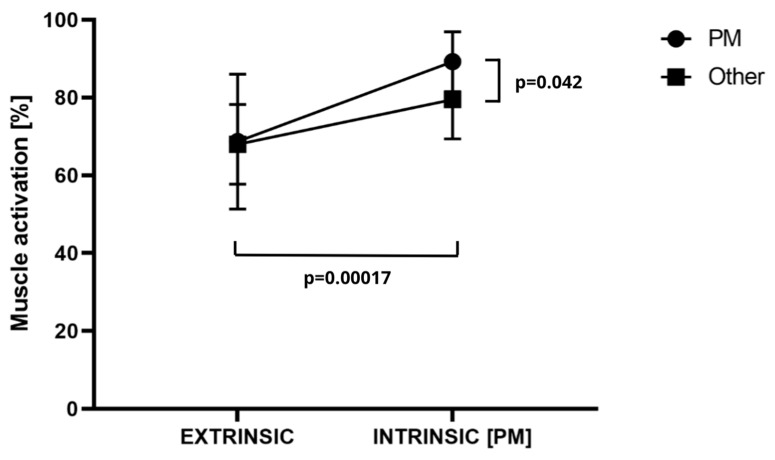
Muscle × extrinsic–intrinsic interaction PM (pectoralis major) activity. *p*-test probability. Other—reference point in the form of the mean of the rest of the muscles outside of the intrinsic focus.

The results of the analysis of variance for PM revealed significant differences for the main effect, specifically for extrinsic–intrinsic (F = 48.90; *p* < 0.0001; η^2^ = 0.59) and for the muscle × extrinsic–intrinsic interaction (F = 5.56; *p* = 0.024; η^2^ = 0.14). No significant differences were identified for the main effect of the muscle (F = 2.95; *p* = 0.095; η^2^ = 0.08). The activation of the PM muscle subsequent to motivation was found to be significantly higher than that observed at extrinsic (68.69 ± 17.39%MVC), with a mean value of 89.23 ± 7.72 %MVC (*p* = 0.00 017; d = 1.37). Activation for the remaining muscles after motivation was significantly higher than that observed for the PM muscle, with a mean value of 78.21 ± 10.53%MVC compared to 68.02 ± 10.21 for the latter (*p* = 0.0018; d = 0.99). The level of activation observed in the PM muscle following motivation (89.23 ± 7.72%MVC) was found to be significantly higher than that observed in the other muscles (78.21 ± 10.53%MVC). This difference was significant (*p* = 0.042; d = 1.13). These results are supported by the data presented in Figure 2.

**Figure 3:**
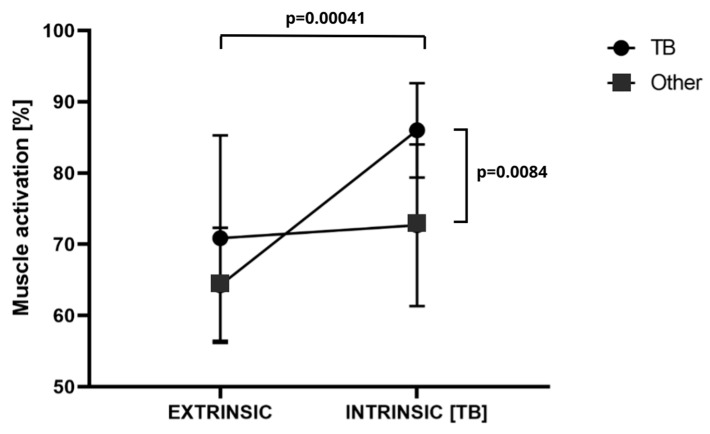
Muscle × extrinsic–intrinsic interaction TB (triceps brachii) activity. *p*-test probability. Other—reference point in the form of the mean of the rest of the muscles outside of the intrinsic focus.

The results of the analysis of variance for TBLONG revealed significant differences for the main effect, specifically for extrinsic–intrinsic (F = 16.83; *p* = 0.0002; η^2^ = 0.33) and for the muscle × extrinsic–intrinsic interaction (F = 12.24; *p* = 0.0013; η^2^ = 0.26). No significant differences were identified for the main effect of the muscle (F = 1.36; *p* = 0.25; η^2^ = 0.038). Activation of the TBLONG muscle after motivation was found to be significantly higher than extrinsic (TBLONG motivation 86.02 ± 6.65%MVC vs. extrinsic 64.27 ± 7.94%MVC; *p* = 0.00041; d = 2.88). No significant differences were observed between the extrinsic (70.93 ± 14.42%MVC) and intrinsic (72.66 ± 11.39%MVC) measurements for the other muscles. The post-motivation activation for the TBLONG muscle (86.02 ± 6.65%MVC) was significantly higher than that for the other muscles (72.66 ± 11.39%MVC; *p* = 0.0084; d = 1.32). These findings are further supported by the data illustrated in the accompanying Figure 3.

## 4. Discussion

The bench press (BP) is one of the most popular upper-body resistance exercises, with numerous variations (e.g., flat, incline, decline) commonly used in practice [29]. The BP is an integral part of resistance training programs used by most athletes to strengthen and gain upper-body muscle mass. The bench press is a compound exercise that involves the pectoralis major of the chest, the anterior deltoids of the shoulder, and the triceps brachii of the upper arm. The bench press is a pushing movement pattern that can change muscle balance for athletes who mainly perform pulling actions in their sport disciplines. The bench press is also a major competitive lift in powerlifting.

The results of the experiment indicate that an intrinsic focus on a specific muscle of the shoulder girdle increases the activity of this muscle during the barbell bench press exercise. The study found that during attentional focus on the activation of the AD, PM and TB muscles, there was a significant increase in the electromyographic activity of this muscle compared to its activity during the bench press (60% 1RM) without attentional focus on its activity (extrinsic motivation). Activation of AD, PM and TB was significantly higher regarding other muscles during the intrinsic motivation set. Furthermore, there were no significant differences between the bench press without additional focus (extrinsic motivation) and the other muscles involved in the bench press while attention was focused solely on the AD, TB and PM muscle activity.

Macharant et al. [15] and Vance et al. [30] investigated the effect of intrinsic focus during exercise of the biceps brachii muscle on an isokinetic dynamometer [15] and during bench press [29]. They showed that intrinsic focus caused a significant increase in the activity of this muscle during resistance exercise, which is similar to our study in which intrinsic focus caused an increase in AD PM and TBLong muscle activation during resistance exercise.

Prior research has shown that it is possible to enhance the activity of the pectoralis major muscle during exercises targeting the chest at loads between 20 and 60% of 1RM, and to augment the activity of the triceps muscle during exercises focusing on the triceps at loads between 20 and 50% 1RM [14,16]. These findings are consistent with our own observations. Schoenfeld and Contreras [12] propose that an increase in muscle activity via internal focus is likely to be less effective at higher external loads, such as 80% 1RM. This is due to the greater recruitment of motor units at higher external loads, which results in a reduction in the number of motor units that can be selectively activated. This may explain the lack of increased muscle activation observed at high external loads.

Rebbeca and Cook [2] indicate that trained individuals did not exhibit increased activation of the pectoralis major muscle or the triceps brachii muscle in response to instructions to focus attention on these muscles. In contrast, Snyder and Fry [14] found that trained individuals were able to increase the activity of the pectoralis major muscle when given instructions to focus attention on the PM muscle, a result that is also supported by our study. However, they were unable to alter the activity of the triceps brachii muscle when provided with specific instructions, which contrasts with the findings of our study.

Snyder and Fry also reported an increase in the activity of the AD when participants were instructed to focus attention on this muscle [14]. This finding is consistent with the results of our study. However, in a study by Rebecca and Cook [2], no changes were observed in the activity of the AD when participants were instructed to concentrate on the activation of this muscle.

The activation of muscles during resistance training has been identified as a reliable indicator of subsequent muscle hypertrophy [31,32]. Consequently, it is beneficial to determine how muscle activity can be modified to achieve the desired training outcome. The findings of our research demonstrate that it is possible to alter muscle activity and thus train individuals by selectively activating specific muscle groups using verbal instructions [33].

In this study, several limitations need to be acknowledged. First, the sample size was relatively small, consisting of only 12 male participants with at least five years of strength training experience. This limits the generalizability of the findings to broader populations, particularly to those with different training backgrounds or to female participants. Second, the study design may have introduced an order effect, as all participants were tested in the extrinsic state first, followed by the intrinsic state. Fatigue occurring during the second phase could have influenced muscle recruitment patterns, potentially affecting the results. Future studies should consider counterbalancing the order of conditions to mitigate this effect. Third, despite providing additional information about the extrinsic state, we cannot fully ascertain the participants’ mental focus during the initial phase. Having read and signed the consent form, participants might have been partially aware of the study’s aims, which could have altered their approach and focus during the extrinsic-only effort. Fourth, the study focused on only three specific muscles—pectoralis major, anterior deltoid, and triceps brachii—during the bench press, potentially overlooking other muscles involved in the exercise. This narrow focus may limit the understanding of the overall muscle activation patterns during different motivational states. Additionally, the study utilized 60% of 1RM as the resistance load. While this provides insight into moderate load training, it may not fully capture muscle activation patterns at higher or lower intensities. Finally, the short-term nature of the study precludes any conclusions about the long-term effects of intrinsic or extrinsic motivation on muscle activation and performance outcomes. Despite these limitations, it is important to note that even with the apparent increase (at least visually) in “Other” muscle activity from the extrinsic to the intrinsic phase—possibly due to fatigue—the interaction effects were detected as significant. The EMG activity in the specific muscle groups of focus during the intrinsic phase appeared to increase more than that of the “Other” muscles within the same phase. This suggests that the observed effects are not solely attributable to fatigue but are indeed linked to the shift in motivational focus.

### Practical Implications

The findings of this study have significant practical applications in resistance training, rehabilitation, and athletic development. Specifically, the enhancement of selective muscle activation through intrinsic motivation—focusing attention on specific muscles during the bench press—offers several key benefits.

- Firstly, in optimizing strength training, athletes can incorporate an intrinsic focus to enhance muscle recruitment in targeted areas. By consciously directing effort toward the anterior deltoid or pectoralis major during the bench press, greater muscle activation can be achieved. This approach could lead to more effective strength gains and hypertrophy outcomes, allowing for the design of specialized programs that emphasize specific muscle groups aligned with an athlete’s sport or personal goals.
- In rehabilitation settings, selective muscle activation is crucial for restoring functional movement patterns, particularly in patients recovering from injuries. Therapists can apply these findings by guiding patients to focus on specific muscles during exercises in the hope of improving the recovery of shoulder girdle function post-injury. Utilizing intrinsic motivation techniques can effectively reactivate weakened or atrophied muscles, potentially accelerating rehabilitation outcomes.
- Finally, incorporating intrinsic focus into an athlete’s long-term development plan can promote a stronger mind–muscle connection. This not only enhances immediate training outcomes but also could improve the athlete’s ability to recruit specific muscles during complex movements. Such improvements are valuable in sports performance contexts where precision and control are essential.

## 5. Conclusions

These findings indicate that athletes and practitioners may be able to modulate muscle activation patterns through conscious attentional focus, which could potentially enhance the effectiveness of resistance training programs. By deliberately focusing on specific muscles, individuals may achieve greater muscle engagement, which may in turn lead to improved strength gains and muscle hypertrophy. This technique is particularly relevant in rehabilitation settings, where targeted muscle activation is crucial for recovery and the restoration of functional movement patterns. Future research should explore the long-term effects of intrinsic focus on muscle development and performance across different populations and exercise modalities.

## Figures and Tables

**Table 1:** Basic descriptive statistics, 95% confidence intervals.

Muscle	n	Extrinsic	Intrinsic
		M ± SD, %MVC (−95% + 95% CI)	M ± SD, %MVC (−95% + 95% CI)
AD	12	71.78 ± 11.13 (64.70–78.85)	88.03 ± 8.84 82.41–93.65
other	24	67.18 ± 13.67 (61.41–72.95)	72.62 ± 11.08 (67.95–77.30)
PM	12	68.69 ± 17.39 (57.64–79.73)	89.23 ± 7.72 (84.32–94.14)
other	24	68.02 ± 10.21 (63.71–72.33)	78.21 ± 10.53 (73.76–82.65)
TBL	12	64.27 ± 7.94 (59.22–69.31)	86.02 ± 6.65 (81.80–90.25)
other	24	70.93 ± 14.42 (64.85–77.02)	72.66 ± 11.39 (67.85–77.48)

M—mean; SD—standard deviation; ±95%, CI—confidence intervals; n—sample size. AD—anterior deltoid, PM—pectoralis major, and TBL—triceps brachii long; other—reference point in the form of the mean of the rest of the muscles outside of the intrinsic focus.

## Data Availability

All data are contained within the manuscript.
